# Gamma tocotrienol targets tyrosine phosphatase SHP2 in mammospheres resulting in cell death through RAS/ERK pathway

**DOI:** 10.1186/s12885-015-1614-1

**Published:** 2015-08-28

**Authors:** Wenyi Gu, Indira Prasadam, Meihua Yu, Fengxia Zhang, Patrick Ling, Yin Xiao, Chengzhong Yu

**Affiliations:** 1 Australian Institute for Bioengineering and Nanotechnology, the University of Queensland, The corner of Cooper Rd. St Lucia, Brisbane, QLD 4072 Australia; 2 Institute of Health and Biomedical Innovation, Queensland University of Technology, Brisbane, Australia; 3 School of Biomedical Science, the University of Queensland, Brisbane, Australia

## Abstract

**Background:**

There is increasing evidence supporting the concept of cancer stem cells (CSCs), which are responsible for the initiation, growth and metastasis of tumors. CSCs are thus considered the target for future cancer therapies. To achieve this goal, identifying potential therapeutic targets for CSCs is essential.

**Methods:**

We used a natural product of vitamin E, gamma tocotrienol (gamma-T3), to treat mammospheres and spheres from colon and cervical cancers. Western blotting and real-time RT-PCR were employed to identify the gene and protein targets of gamma-T3 in mammospheres.

**Results:**

We found that mammosphere growth was inhibited in a dose dependent manner, with total inhibition at high doses. Gamma-T3 also inhibited sphere growth in two other human epithelial cancers, colon and cervix. Our results suggested that both Src homology 2 domain-containing phosphatase 1 (SHP1) and 2 (SHP2) were affected by gamma-T3 which was accompanied by a decrease in K- and H-Ras gene expression and phosphorylated ERK protein levels in a dose dependent way. In contrast, expression of self-renewal genes TGF-beta and LIF, as well as ESR signal pathways were not affected by the treatment. These results suggest that gamma-T3 specifically targets SHP2 and the RAS/ERK signaling pathway.

**Conclusions:**

SHP1 and SHP2 are potential therapeutic targets for breast CSCs and gamma-T3 is a promising natural drug for future breast cancer therapy.

**Electronic supplementary material:**

The online version of this article (doi:10.1186/s12885-015-1614-1) contains supplementary material, which is available to authorized users.

## Background

The concept of cancer stem cells (CSCs) describes that tumors contain a small proportion of self-renewing and pluri-potent cells that are responsible for initiating and maintaining tumor growth [1]. This concept is well established in leukemia and has also been reported in a few solid tumor types [2–4]. Recent studies further confirm that a specific cell population is responsible for the initiation and growth of solid tumors [5–7]. These cells usually express high levels of multiple drug resistant gene (MDR1) [8] and ATP binding cassette (ABC) transporter [9] and are therefore resistant to chemotherapy and considered as the major source of drug-resistance in tumors. Moreover, it has been demonstrated that CSCs are responsible for metastasis [7, 10, 11], which is another major cause of cancer-related death. CSCs are thus regarded as an essential target for future advanced cancer therapy.

To achieve the goal of effective treatment of CSCs, identifying specific therapeutic targets is vital. Apart from high throughput screening methods such as microarrays, identifying novel targets of inhibitors or natural drugs is an alternative. A few natural compounds are reported to have inhibitory effects on CSCs [12–14]. These products are valuable for future CSC targeted therapy as they are normally less toxic than chemotherapeutic drugs. For example, vitamin E isotype gamma tocotrienol (γ-T3) was shown to be effective at inhibiting cancer cell growth in several solid tumor models through apoptosis or cell stress related pathways [15–18]. In CSCs, Ling and colleagues reported that γ-T3 could effectively inhibit CSC growth in prostate cancer *in vitro* and *in vivo* [19]. They also showed that the CD44 expression of the CSCs was decreased by γ-T3 treatment. CD44 is one of the important epithelial CSC markers, suggesting γ-T3 may affect the stemness of prostate CSCs. However, the detailed mechanism of how γ-T3 suppresses CD44 expression and prostate CSC growth remains unknown. In addition, it is still not clear whether the reduction of CD44 expression was through γ-T3 directly interacting with CD44 or through an indirect interaction with other molecules.

Previously, a study reported that γ-T3 inhibited STAT3 phosphorylation and JAK/STAT pathway activation in different melanoma cell lines, resulting in apoptosis of the cancer cells [20]. This inhibition was through the induction of SHP1 expression by γ-T3, suggesting that SHP1 was a target of γ-T3 [20]. However, whether this is the case in CSCs has not been reported or whether there are any new targets in JAK/Stat pathway for γ-T3 remains unknown. In JAK/Stat pathway, there are two very closely related proteins SHP1 and SHP2, they share highly similar structures and sequences. Both of them have two Src homology 2 domains (SH2) that bind to several tyrosine-phosphorylated proteins [21–23]. For biological function, however, SHP1 plays a dominant negative regulation role in the pathway [24, 25] while SHP2 plays a major positive role [26–28]. Phosphorylation of SHP2 activates associate proteins such as Grb2 and Gab2 and form a protein complex SHP2/Grb2/Gab2. This complex then activates the downstream target RAS and other components of the RAS/ERK pathways [29–31]. SHP2 is encoded by PTPN11, a proto-oncogene in hematologic cells [32]. Mutation of PTHN11 has been associated with juvenile myelomonocytic leukemias, neuroblastoma, melanoma, acute myeloid leukemia, breast cancer, lung cancer, and colorectal cancer [33]. SHP2 protein levels are elevated in some cancers including cervical cancer [34] and approx 72 % of breast cancer cell lines [35]. Many cell types express SHP2 however, SHP1 is expressed in a restricted number of cell types [36, 37]. These data indicate that compared with SHP1, SHP2 is more likely to be an onco-protein involved in cancer development. Indeed, several studies have shown that inhibition of SHP2 can retard cancer cell growth [38]. Inhibition of SHP2 gene expression with shRNA was also associated with cell transformation from mesenchymal to epithelial cells, indicating a promoting role of SHP2 in carcinogenesis [39]. A recent study has shown that SHP2 plays an essential role in the initiation, progression, and metastasis of breast cancer by activating stemness-associated transcription factors such as c-Myc and ZEB1 [40], further demonstrating its oncogenic role in cancer stem cells. However, there is no report on if γ-T3 targets SHP2 in any cancer types.

Breast cancer is the leading cause of cancer related death among women. Though some studies have shown that using γ-T3 can effectively induce apoptosis or cell cycle arrest in breast cancer cells [41, 18], there is no report on γ-T3 treating breast CSCs. Particularly, there is no report exploring the potential targets of SHP1 and SHP2 in breast CSCs. In this study, we have demonstrated that γ-T3 had a broad inhibitory effect on human epithelial CSCs including those from breast, colon, and cervical cancers. We found that apart from the effect on SHP1, γ-T3 also targeted SHP2 in breast cancer and that the γ-T3 inhibitory effect on CSC growth was through the RAS/ERK pathway. Moreover, we report here that the level of phosphorylated SHP2 protein increases in breast CSCs, compared with their parental cancer cells, suggesting that SPH2 may play an important role in breast CSC growth and may be considered as a potential therapeutic target for breast CSCs.

## Methods

### Cell lines and mammosphere culture

This study did not involve in human subjects including human material or human data. Epithelial cancer cell lines of breast cancer MCF-7 (ATCC, HTB-22™), colon cancer HCT-116 (ATCC, CCL-247™), and cervical cancer HeLa (ATCC, CCL-2) were purchased from ATCC (during years 2010–2012) and maintained in complete Dulbecco’s Modified Eagles Medium (DMEM, Invitrogen, Australia) as previously described [42]. The sphere culture medium was prepared as previously reported [43]. For the first passage of sphere culture, 1 × 10^4^ cancer cells were seeded into a T25 flask with 6 ml of sphere culture medium. Cells were cultured in suspension for 4 days at 37 °C with 5 % CO_2_ and an additional 2.5 ml of sphere culture medium was added to the culture. The culture continued for another 3–4 days and spheres were harvested by centrifugation at 300 × g for 3 min and their numbers were counted after re-suspension in 2–5 ml medium. For the second and following passage sphere culture, the spheres were treated with 1:1 diluted 2.5 % Trypsin-EDTA (Invitrogen, Australia) for 5 min at 37 °C and washed with sphere culture medium. Sphere cells were separated by repeating pipetting. The separated cells were passed through a cell strainer (40 μM, BD, Australia) and were counted for continuous sphere culture or other assays in low-adherence 6-well plates (Sigma-Aldrich, Australia) or T25 flasks.

### γ-T3 treatment, flow cytometry, and fluorescence microscopy

Highly purified γ-T3 was provided by Davos Life Science Pte. Ltd, Singapore and was prepared as a stock solution in 100 % ethanol at a concentration of 20 mg/ml and stored at −20 °C freezer. The stock solution was diluted in ethanol into 2 mg/ml and added directly into the sphere cultural medium at different concentrations. The control group was added with same amount of ethanol. The cells were cultured for 7–8 days until spheres formed. For FACS analysis, cells were harvested, dispersed from spheres and stained with antibodies to CD44 or CD133 conjugated with FITC and CD24 conjugated with RPE (Invitrogen, Australia) at concentrations of 1:100 (V/V, 5 × 10^5^ cells). The cells were washed 2 times with 1 % fetal calf serum (FCS)/PBS then fixed with 2 % paraformaldehyde/PBS for FACS analysis using Calibur or FACS Canto (BD, Australia).

### Immuno-blotting

Immune-blotting analysis was conducted as previously described [42]. The separated cells from mammospheres and cell lysates were prepared in RIPA buffer after γ-T3 treatment. Before total protein measurement, the samples were sonicated briefly. Anti-human β-tubulin antibody was from Sigma-Aldrich. Anti-pERK antibody was from Cell signaling. Rabbit anti-SHP2 antibody was from Sigma-Aldrich and rabbit anti-SHP2 (pS576) polyclonal antibody was from Invitrogen USA. Mouse anti-SHP1 antibody was purchased from Cell Signaling Technology.

### Real-time RT-PCR

Total RNA extraction from mammosphere cells was prepared as instructed by the manufacturer using TRIzol® reagent (Invitrogen, Australia). Reverse transcription reactions were performed with oligo-dT primer using the High Capacity cDNA RT Kit (Applied Biosystems). Real-time PCR was carried out with SYBR green master mixture (Promega) on a Rotor-Gene RG-3000 (Corbett Research, Australia) with the program pre-heating 95 °C 10 min; then 40 cycles of 94 °C 15 s; 58 °C 30 s; and 72 °C 45 s. The primers were: TGF-β-1: F 5′-CAACAATTCCTGGCGATACC, R 5′-GAACCCG TTGATGTCCACTT; TGF-β-2: F 5′-GAGTGCCTGAACAACGGATT, R 5′-TGCAGCAGGGACA GTGTAAG; TGF-β-3: F 5′-GCAACTTGGAGGAGAACTGC, R 5′-CTGTGGGTTGTGTCTGC ACT; LIF: F 5′-CCCTGTCGCTCTCTAAGCAC, R 5′-ATCCTGGACAAGGGTGAGTG; H-Ras: F 5′-GTGGTCATTGATGGGGAGAC, R 5′-ACGTCATCCGAGTCCTTCAC; K-Ras: F 5′-TGT CAAGCTCAGCACAATCTG, R 5′-GGTAGGGAGGCAAGATGACA; ERBB2: F 5′-GACATTGACGAGACAGAG, R 5′-ACACAGTCACACCATAAC; ESR1: F 5′-CACATCAGGCACATGAGTAACAA, R 5′-TCCAGCAGCAGGTCATAGAG; SHP1: F 5′ TTTCAAGAAGACGGGGATTG, R 5′ CGGACTCCTGCTTCTTGTTC; SHP2: F 5′ AGAGCCACCCTGGAGATTTT, R 5′ CTCCTCCACCAACGTCGTAT. The internal control was *18S rRNA* (F: 5′-CCATCGAACGTCTGCCCTA; R: 5′-TCACCCGTGGTCACCATG) is used to normalize target gene expression.

### Data analysis

Besides technical repeats (3 repeats) of real-time RT-PCR, biological repeats (3 times) were performed. Data collected from each (experimental and control) group were expressed as mean ± SD. The one-way ANOVA and unpaired *t*-test (GraphPad Prism 6 program) were used to analyze the differences between groups and discriminate the significant differences (two-tails, *P* < 0.05) between experimental and control groups.

## Results

### Mammosphere cells and treatment with γ-T3

**Fig. 1:**
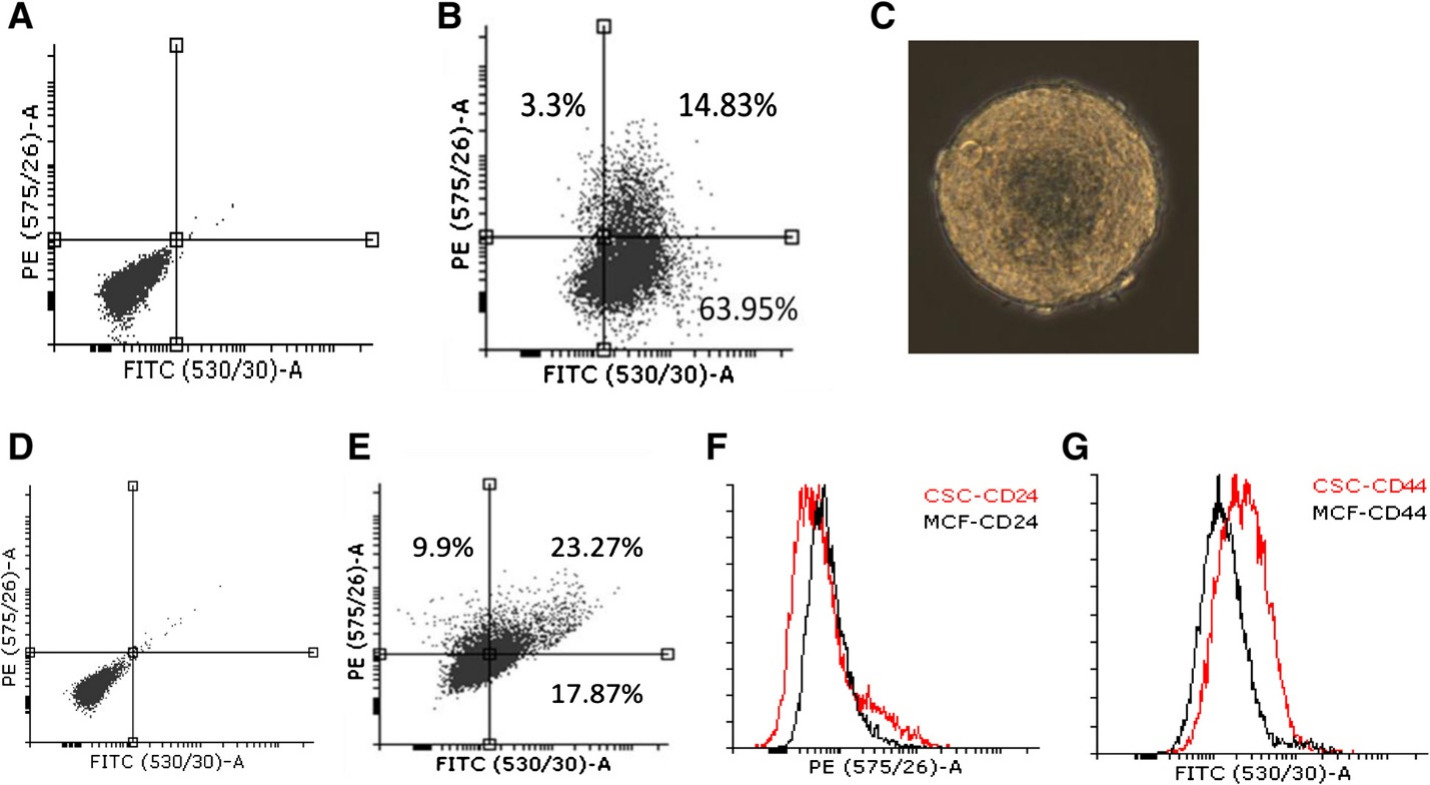
CD44 and CD24 expressions of mammosphere and MCF-7 cells: Mammosphere cells and their parental MCF-7 cells were labeled for CD44 and CD24 expression. **a** antibody isotype controls of mammosphere cells. **b** mammosphere cells stained with CD44 and CD24 antibodies conjugated with FITC and PE respectively. **c** an image of a mammosphere cultured for 8 days, the compacted sphere contains numerous cells. **d** MCF-7 cells stained with isotype controls. **e** MCF-7 cells stained with CD44 and CD24 antibodies conjugated with FITC and PE respectively. **f** and **g** histogram diagrams to show the CD24 (**f**) and CD44 (**g**) expression shifts (the decrease of CD24 and the increase of CD44 in mammosphere cells)

**Fig. 2:**
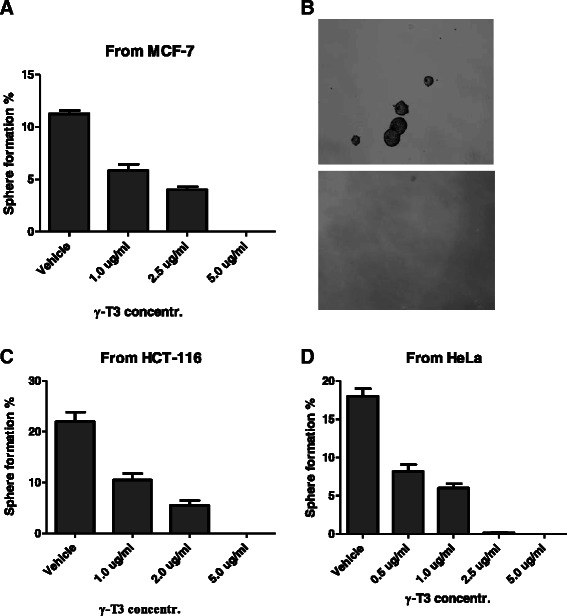
Dose-dependent inhibition of sphere growth of three epithelial cancers by γ-T3: **a** γ-T3 inhibited mammosphere formation and growth from MCF-7 cells in a dose-dependent manner from 1.0 to 5.0 μg/ml. **b** morphology of mammosphere in vehicle control and in 5 μg/ml G-T3 treatment. **c** and **d** In addition to breast cancer, γ-T3 inhibited sphere formation of colon (HCT-116) and cervical (HeLa) cancers respectively

We used the reported sphere culture method [43, 44] to culture mammospheres from breast carcinoma cell line MCF-7. The mammosphere formation rate was about 20 % from the cell line in several generations (passages) of culture. The mammosphere cells were separated and characterized for surface expression of CD44 and CD24, which are commonly considered as markers of breast cancer stem cells [2]. The results showed CD44 expression increased in mammosphere cells and the positive cell proportion increased from 5.62 % of parent MCF-7 cells to 45.65 % of mammosphere cells (Fig. 1). Meanwhile, the expression of CD24 alone and CD24/CD44 double positive cells was deceased from 15.58 to 5.99 % and from 18.99 to 12.18 %, respectively (Fig. 1). These profiles suggest that after sphere culture the mammosphere cells are enriched for breast cancer stem-like cells, which is consistent with previous reports [2, 45]. We then treated mammosphere cells with γ-T3 in sphere culture by adding γ-T3 directly into the sphere culture medium. As shown in Fig. 2, γ-T3 exhibited a dose-dependent inhibition of the growth of mammosphere cells and sphere formation from MCF-7 cells. At 5 μg/ml, the sphere growth was totally inhibited (Fig. 2a).

### γ-T3 inhibits spherical cell growth of other epithelial cancers

To extend the above observations in breast cancer to other epithelial cancers, we isolated sphere forming cells (SFCs) from cervical (HeLa cells) and colon (HCT-116 cells) cancers using the same sphere culture method and analyzed their surface markers. The results showed that these SFCs expressed specific markers as expected (Additional file 1: Figure S1) which were consistent with previous reports [44, 46], suggesting that after sphere culture, CSCs are enriched in both cancer cell lines. We treated these spherical cells with γ-T3. Similar growth inhibition to mammospheres was observed for these two cancers (Fig. 2c and d), revealing that γ-T3 has a broad spectrum of inhibition effect on the growth of CSCs in epithelial cancers, including prostate cancer as reported previously [19]. In addition, at a dose of 5.0 μg/ml the growth of CSCs was totally inhibited (Fig. 2). It is also noted that comparisons between the three epithelial cancers showed, cervical CSCs from HeLa cells were slightly more sensitive to γ-T3 than CSCs from MCF-7 and HCT-116 (Fig. 2).

### γ-T3 targets SHP2 and also increases SHP1 protein levels in mammosphere cells

**Fig. 3:**
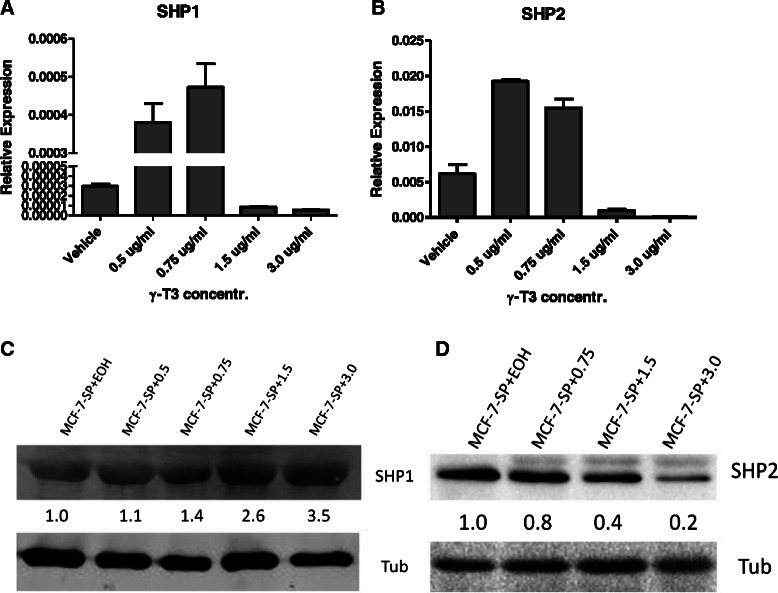
SHP 1 and SHP2 gene and protein expressions after γ-T3 treatment. **a** and **b** real-time PCR results showing SHP1 and SHP2 mRNA expression in mammosphere cells treated with different doses of γ-T3. **c** and **d** Western blotting results showing SHP1 and SHP2 protein expression levels after γ-T3 treatment at different doses. *EOH* ethanol, *SP* sphere cells, *Tub* human β-tublin

**Fig. 4:**
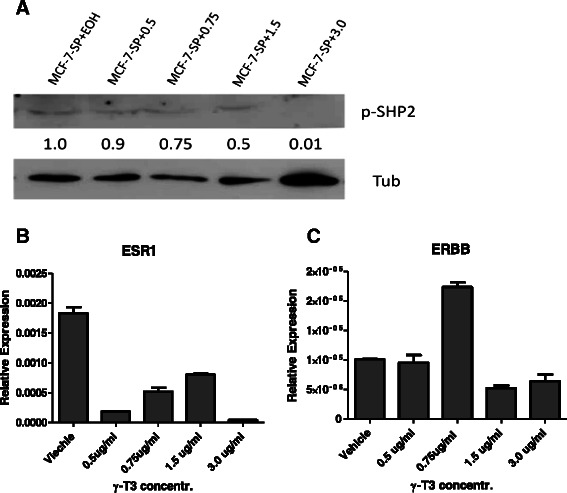
Phosphorylated SHP2 and other related gene expression after γ-T3 treatment. **a** Western blotting result shows phosphorylated SHP2 (p-SHP2) protein levels in mammosphere cells after γ-T3 treatment with different doses. **b** and **c**, real-time RT-PCR results show mRNA expressions of ERBB and ESR in γ-T3 treated mammosphere cells. *EOH* ethanol; *SP* sphere cells; *Tub* human β-tublin

To test whether SHP1 and/or SHP2 are affected by γ-T3 treatment, we firstly measured mRNA levels of SHP1 and SHP2 in γ-T3 treated mammosphere cells. SHP1 gene expression did not increase in a dose dependent way as γ-T3 dose increased (Fig. 3a). But as we expected, SHP2 gene expression did decrease in a dose dependent manner (Fig. 3b). To confirm this in protein levels, we measured total protein levels of SHP1 and SHP2 in γ-T3 treated mammosphere cells. As shown in Fig. 3c and d, SHP1 levels increased while SHP2 levels decreased as γ-T3 dose increased. To further prove that SHP2 biological function was inhibited by γ-T3 treatment, we also measured phosphorylated SHP2 protein (p-SHP2) in γ-T3 treated cells, the result showed that p-SHP2 also decreased as γ-T3 dose increased (Fig. 4a). To further explore other possible targets of γ-T3, we measured the expression of other two closely related genes, ERBB2 and ESR1. ERBB2 is also known as HER 2, and is a trans-membrane receptor protein and an up-stream activator of SHP2 and RAS-ERK pathway [47]. ESR1 is an estrogen receptor which can be associated with the cell surface membrane and after activation can be involved in the activation of MAPK [48]. However, our data indicated that these two gene expressions were not affected by γ-T3 treatment (Fig. 4b and c). Therefore, we focused on SHP2 protein in this study.

### Dose-dependent down-regulation of RAS/ERK pathway by γ-T3

**Fig. 5:**
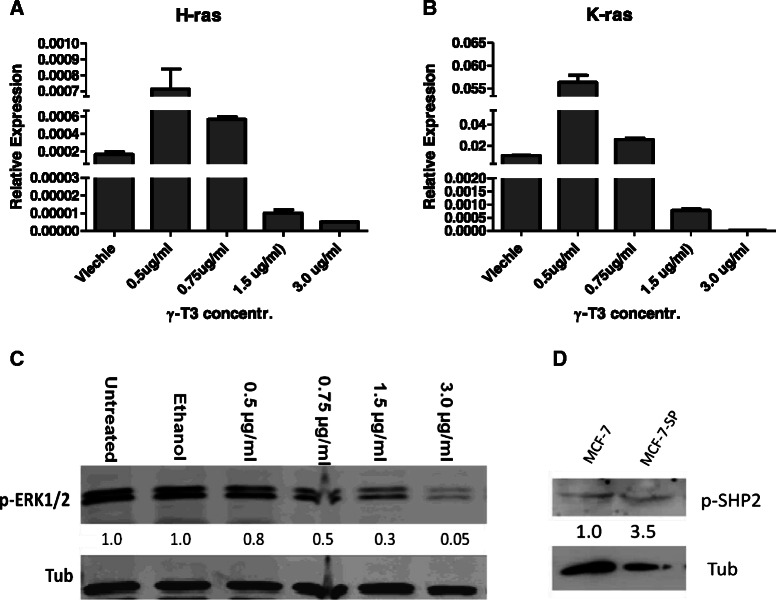
Ras/ERK signal pathway is down-regulated after γ-T3 treatment. As components of SHP2/RAS/ERK pathway, Ras gene expression including H-RAS (**a**) and K-RAS (**b**) was decreased as γ-T3 dose increased from 0.5 to 3.0 μg/ml. **c** ERK protein levels decrease as γ-T3 dose increases. **d** Western result shows the comparison of p-SHP2 protein level in MCF-7 cells and mammosphere cells (MCF-7-SP). *Tub* human β-tublin

SH2-domain containing proteins, like SHP2, when phosphorylated on tyrosine residues, culminate in the activation of a number of intracellular signalling cascades including the STAT5, Ras/mitogen activated protein kinase (MAPK) and phosphoinositide-3 kinase (PI3K) pathways [49]. In this singling pathway Ras is a direct downstream component of SHP2, hence we assumed that the down-regulation of SHP2 would also affect Ras gene expression. In addition, the increased SHP1 protein expression by γ-T3 could also affect RAS/ERK pathway through negatively regulating JAK/Stat pathway. We thus measured Ras gene expression in γ-T3 treated cells. As we expected, both H-Ras and K-Ras gene expressions decreased as γ-T3 doses increased (Fig. 5a and b). To further testify that γ-T3 targeted SHP1 and SHP2 and through RAS/ERK pathway, we also measured another downstream component, the phosphorylated ERK protein in γ-T3 treated cells and we found that phosphorylated ERK decreased in γ-T3 treated cells in a dose-dependent manner (Fig. 5c). All these results together suggest that SHP1, SHP2, and RAS/ERK pathways have been affected by γ-T3 treatment. To understand if SHP2 is more activated in mammosphere cells, we compared p-SHP2 level in MCF-7 and mammosphere cells (Fig. 5d). The result indicated that an increased p-SHP2 was observed in mammosphere cells.

### γ-T3 did not affect self-renewal pathways in breast CSCs

**Fig. 6:**
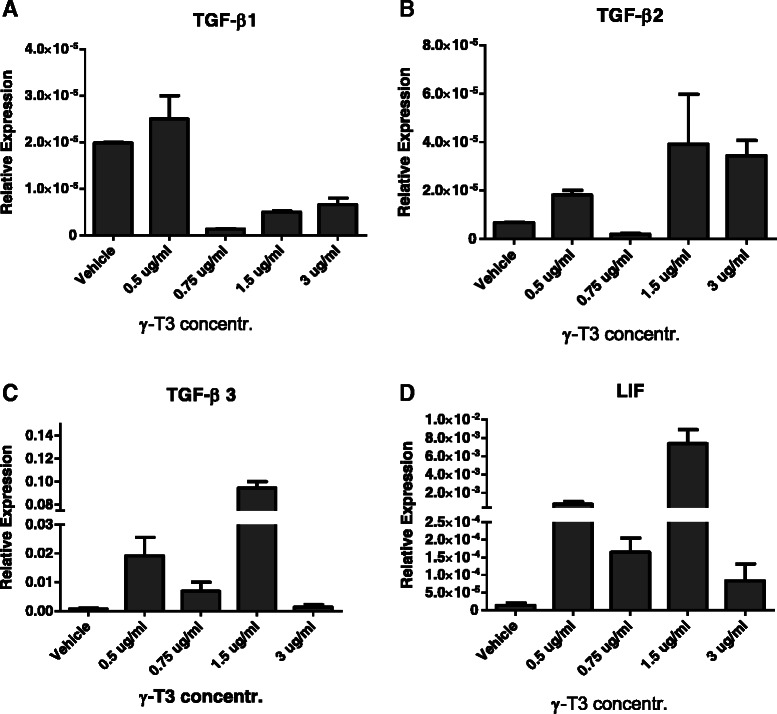
Self-renewal genes of TGF-β and LIF expressions in mammosphere cells after γ-T3 treatment. Self-renewal gene expression of TGF-β (including β1 to β3, **a** to **c**) and LIF (**d**) mRNA levels were not affected by γ-T3 treatment in a dose dependent way, suggesting these pathways may not be involved in γ-T3 induced cell death

**Fig. 7:**
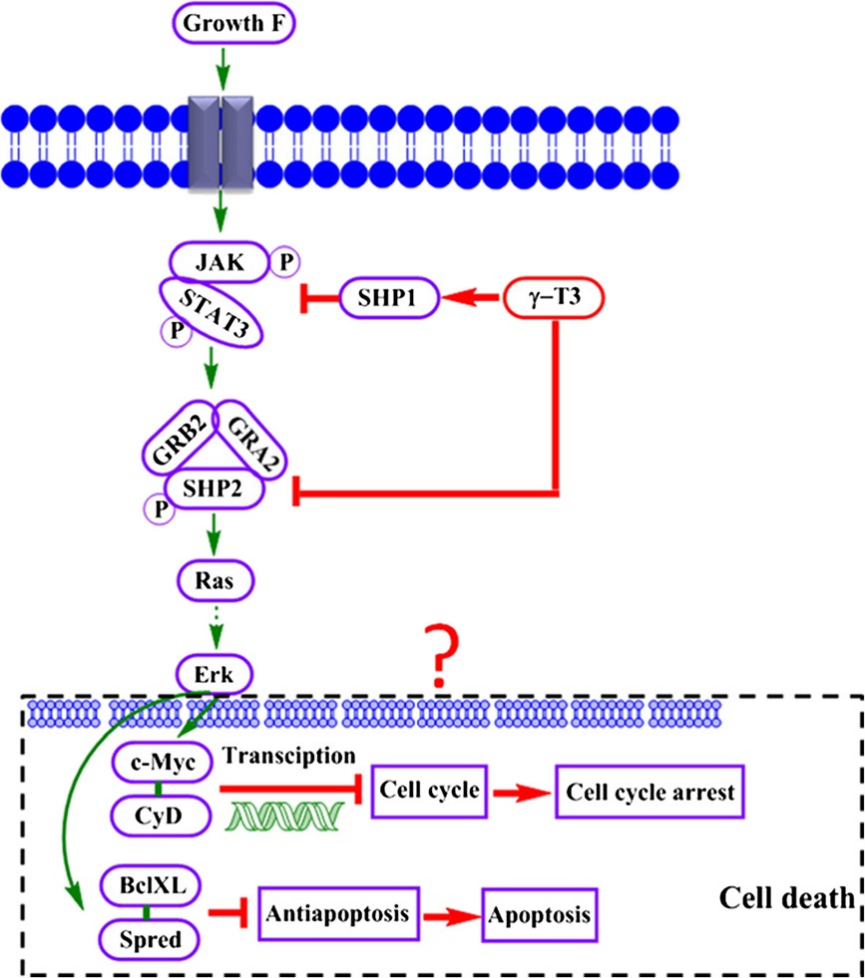
A schematic diagram to show SHP/RAS/ERK pathway and γ-T3 targets in mammosphere cells. In mammosphere cells, γ-T3 targets both SHP1 and SHP2 to down regulate RAS/ERK pathway and subsequently leads to the cell death in mammospheres. For SHP1, γ-T3 promotes its protein production to down-regulate Ras/ERK pathways. γ-T3 through down-regulating the protein level and phosphorylation of SHP2 reduces the interaction with associated proteins such as GRB2 and GAB2, leading to activation of the RAS/ERK pathway. It is postulated that in the nucleus, the double action of down-regulation of ERK interferes with the transcription factors C-myc and Cyd ensuring normal cell cycles (*green arrows*) and the anti-apoptosis factors Bcl-XL and Spred subsequently cause cell cycle arrest and apoptosis of the mammosphere cells (*Red arrows*)

It has been shown that TGF-β and LIF are two important self-renewal genes for stem cells [50, 51]. In cervical cancer, we showed that silencing human papillomavirus oncogenes E6/E7 with shRNA delivered by lentiviral vector in cervical CSCs could lead to the down regulation of self-renewal gene TGF-β [44]. To investigate if γ-T3 also inhibits sphere growth through impairing CSC self-renewal ability by down-regulating TGF-β and LIF, we measured gene expression in MCF-7 and mammosphere cells. However, the results failed to show any dose-dependent reduction of gene expressions (Fig. 6). To further confirm this, we also measured gene expression in HeLa sphere cells treated with γ-T3 (Additional file 1: Figure S2). Similar results were obtained in HeLa sphere cells, suggesting that γ-T3 inhibits CSC growth was not through inhibiting their self-renewal ability via TGF-β and LIF pathways. This result also suggests that γ-T3 may specifically target SHP1/SHP2/RAS/ERK pathways and which probably leads to the inhibition of C-Myc and CyD transcription factors and anti-apoptosis factor such as Bcl-XL and Spred resulting in cell cycle arrest and apoptosis of mammosphere cells (Fig. 7).

## Discussion

In this study, based on the evidence that γ-T3 could effectively inhibit sphere growth in three different epithelial cancers, we believed that its target(s) would be vital for self-renewal and proliferation of CSCs and thus tried to identify them. A previous study showed that in skin cancer U266 and SCC4 cells, γ-T3 inhibited cell growth through induction of SHP1 and suppression of its related JAK/STAT/RAS/ERK signaling pathway [20]. We thought SHP1 could be also a target of γ-T3 in CSCs. In addition, as SHP2 shares two SH2 domains with SHP1 but has opposite biological functions and is widely expressed in many cell types compared to the restricted expression of SHP1, we thought SHP2 could be a target of γ-T3 as well. Indeed, our results proved that besides increasing SHP1 protein levels, γ-T3 also targeted PNPT11/SHP2 and inhibited their expression in mammosphere cells in a dose dependent manner. As a consequence of the dual effects of γ-T3, the downstream RAS/ERK signal pathway was down-regulated and this further led to the possible cell cycle arrest and apoptosis of mammosphere cells (Fig. 7). In contrast, other unrelated genes/pathways such as ERBB2, ESR1, and TGF-β were not affected, indicating that SHP1 and SHP2 are specific targets of γ-T3 in at least mammosphere cells. As γ-T3 also effectively inhibits the spherical cell growth of colon and cervical cancers, we assume their SHP1 and 2 had been affected. To our knowledge, the study of the effect of γ-T3 on both SHP1 and SHP2 in mammosphere cells has not been reported before. Particularly, the down-regulation of PNPT11/SHP2 in mammospheres by γ-T3 is a new finding. The implication of this study is that SHP2 may serve as a target for breast CSC therapy in the future. Our study also provides evidence that γ-T3 can be considered as a promising therapeutic candidate to target both SHP1/SHP2 in breast CSCs. As treating breast cancer cells (eg. MCF-7) normally needs a dose ranged from 10 to 40 μg/ml, if this dose is applied in clinical settings, it will be sufficient to totally inhibit breast CSC growth according to our present data.

Our results are consistent with a recent study in which the authors demonstrated that SHP2 played a fundamental role in the initiation, progression, and metastasis of human epidermal growth factor receptor 2 (HER2)-positive and triple-negative breast cancers [40]. They showed that knockdown of SHP2 eradicated breast tumour-initiating cells in xenograft models, and SHP2 depletion prevented invasion in three-dimensional cultures and in a transductal invasion assay *in vivo*. In addition, SHP2 knockdown in established breast tumours blocked their growth and reduced metastasis [40]. They also showed that these effects of SHP2 were through activation of stemness-associated transcription factors, including v-myc myelocytomatosis viral oncogene homolog (c-Myc) and zinc finger E-box binding homeobox 1 (ZEB1). C-Myc is also a downstream component of Ras/ ERK pathway, indicating their result is consistent with our data. In addition, in the study they showed that depletion of SHP2 could impair self-renewal ability of breast cancer cells by using sphere culture method [40]. This is obviously confirmed by our current study. However, in our study we further demonstrated that this was not through TGF-β and LIF pathways (Fig. 6). All data above support our conclusion and further prove that SHP2 provides a rationale target for breast CSC therapy and γ-T3 should be considered as a good candidate drug for the therapy.

SHP2 is a member of the non-receptor protein-tyrosine phosphatase family and its cell survival, proliferation, migration, and differentiation functions have been well characterized in hematopoietic cells and hematopoietic stem cells (HSCs). The loss of Ptpn11 in murine hematopoietic cells can lead to bone marrow aplasia and lethality [36]. Mutant of Ptpn11 gene in mice show a rapid loss of HSCs and immature progenitors of all hematopoietic lineages in a gene dosage-dependent and cell-autonomous manner. Ptpn11-deficient HSCs and progenitors undergo apoptosis concomitant with increased Noxa expression. Mutant HSCs/progenitors also show defective ERK and Akt activation in response to stem cell factors and diminished thrombopoietin-evoked ERK activation [36]. Over-expression of SHP2 was associated with leukemogenesis in adult human leukemia [52]. Thus, Shp2 plays a critical role in controlling the survival and maintenance of HSCs and immature progenitors *in vitro* and *in vivo*.

SHP2 has been related to oncogenesis because like Src (a well-known oncogene), PTNT11 is also reported as a proto-oncogene [32]. It has been reported that the mutation of PTPN11 is associated with high prevalence of juvenile myelomonocytic leukemias, neuroblastoma, melanoma, acute myeloid leukemia, breast cancer, lung cancer, and colorectal cancer [33]. Moreover, SHP2 protein is elevated in some cancers including cervical cancer which is high associated with HPV infection [34]. In breast cancer, 72 % cancer cell lines have increased amounts of the SHP2 protein and dominant-negative SHP2 blocked the growth of breast cancer cells [35]. Furthermore, overexpression of SHP2 appears to have a positive relationship to HER2 overexpression, nuclear accumulation of hormone receptors, higher tumour grade and lymph node metastasis; which suggests that SHP2 promotes breast oncogenesis [35]. In addition, SHP2 promotes HER2-induced signalling and transformation at least in part by dephosphorylating a negative regulatory autophosphorylation site [35, 40]. However we showed that γ-T3 induced SHP2 reduction did not affect ERBB2/HER2 at mRNA levels. Further investigations of protein levels are needed. These results suggest that SHP2 might serve as a therapeutic target against breast cancer and other cancers characterized by ERBB2/HER2 overexpression [53]. However, SHP2 and its potential role in CSCs of solid tumors have not been studied or reported much. In this study, we showed that like in HSCs, p-SHP2 was expressed at higher levels in mammosphere cancer cells compared with parental MCF-7 cells, suggesting that it may play an important role in breast CSCs. This observation can be extended to the spherical cells of two other epithelial tumors, colon and cervix, which showed a similar mammosphere inhibition, however further experimental data are needed to prove this.

## Conclusion

Apart from having confirmed the therapeutic value of γ-T3, this study has also demonstrated that SHP2 is a potential biomarker and therapeutic target for breast CSCs. In addition, it seems that this target may be widely distributed in other epithelial CSCs including colon, cervical and probably prostate cancers. From the therapeutic point of view, γ-T3 has the potential to be an effective drug to target both differentiated cancer cells and CSCs, which is better than conventional chemotherapeutic drugs that only target differentiated cancer cells.

## Additional file

Additional file 1: Figure S1. Characterization of sphere cell forming cells cultured from colon cancer cell line HCT-116. **Figure S2.** Self-renewal gene expressions of γ-T3 treated HeLa spherical cells. (DOCX 167 kb)

## Abbreviations

CSCs: Cancer stem cells
SHP1 and 2: Src homology 2 domain-containing phosphotase 1 and 2
SFCs: Sphere forming cells
TGF-β: Beta transformation growth factor
LIF: Leukemia inhibitory factor
ERBB2: V-erb-b2 avian erythroblastic leukemia viral oncogene homolog 2
ESR1: Estrogen receptor
