# Supplementary material for: Gamma tocotrienol targets tyrosine phosphatase SHP2 in mammospheres resulting in cell death through RAS/ERK pathway
Source: BMC Cancer. 2015 Aug 28;15:609. doi: 10.1186/s12885-015-1614-1 (PMC4552156; doi:10.1186/s12885-015-1614-1)
Supplement: Additional file 1: Figure S1. — Characterization of sphere cell forming cells cultured from colon cancer cell line HCT-116. Figure S2. Self-renewal gene expressions of γ-T3 treated HeLa spherical cells. (DOCX 167 kb) [file 12885_2015_1614_MOESM1_ESM.docx]

**Figure S1: Characterization of sphere cell forming cells cultured from colon cancer cell line HCT-116.**

A

B

C

**Fig S1**: Spherical cells from HCT-116 colon cancer cells were stained for their surface markers. A shows the results of CD133 staining results and antibody isotype control between HCT-116 and SFCs. B shows the CD44 staining results, though the fluorescent intensity of SFCs may not increase but the cell number increased, especially in UR region. C shows the CD24 and CD44 staining results in HeLa (HeLa) and HeLa spherical cells (HeLa-SFC).

**Figure S2: Self-renewal gene expressions of γ-T3 treated HeLa spherical cells.**













**Fig S2:** self-renewal gene expressions of TGF-beta including TGF-β1, β2 and β3 and LIF were measured in HeLa spherical cells treated with different doses of gamma-T3. EOH: ethanol and was used vehicle control. HeLa cells (HeLa) without any treatment was also included as a control.
